# Association of Accelerometer-Measured Light-Intensity Physical Activity with Cardiovascular Diseases: A Large Cohort Study

**DOI:** 10.34133/hds.0470

**Published:** 2026-08-12

**Authors:** Lihui Zhou, Zhi Cao, Yujia Lv, Hongxi Yang, Dun Li, Jing Lin, Chenjie Xu, Yaogang Wang

**Affiliations:** ^1^ Department of Epidemiology and Biostatistics, School of Public Health, Tianjin Medical University, Tianjin, China.; ^2^ School of Public Health, Hangzhou Normal University, Hangzhou, China.; ^3^ School of Public Health, Zhejiang University School of Medicine, Hangzhou, China.; ^4^ Department of Bioinformatics, School of Basic Medical Sciences, Tianjin Medical University, Tianjin, China.; ^5^ School of Integrative Medicine, Public Health Science and Engineering College, Tianjin University of Traditional Chinese Medicine, Tianjin, China.; ^6^ School of Nursing, Tianjin Medical University, Tianjin, China.; ^7^ National Institute of Health Data Science at Peking University, Peking University, Beijing, China.

## Abstract

**Background:** Light-intensity physical activity (LPA) constitutes a substantial portion of daily activities; however, it is more challenging to record and measure accurately compared to moderate- to vigorous-intensity physical activity (MVPA). Consequently, the health benefits associated with LPA are frequently overlooked. Our study aimed to investigate the associations of LPA pattern including objectively measured LPA’s duration, proportion, and timing with risk of incident cardiovascular diseases (CVDs). **Methods:** With the UK Biobank, 83,658 participants free of CVD with valid accelerometry data were included between 2013 and 2015. Data on physical activity (PA) were collected using the Axivity AX3 wrist-based triaxial accelerometer worn for 1 week. LPA duration was estimated using machine learning models based on bouts data from wrist-worn accelerometers. The LPA proportion was calculated by dividing the amount of LPA by the sum of LPA and MVPA. LPA timing was determined using hierarchical clustering analysis based on the average duration of LPA within each time window across the 24-h cycle. CVDs of interest including coronary heart disease (CHD), stroke, heart failure (HF), and atrial fibrillation (AF) were identified through the linkage to hospital admission records and death registers. **Results:** Over a median follow-up duration of 7.98 (interquartile range: 7.43 to 8.47) years, a total of 2,239 CVDs, including 1,014 CHDs, 352 strokes, 796 AFs, and 194 HFs, were recorded. An L-shaped relationship was found between LPA duration and the risk of HF. Compared to individuals in the lowest quartile, those in the second (hazard ratio [HR], 0.64; 95% confidence interval [CI]: 0.44, 0.93), third (HR, 0.56; 95% CI: 0.37, 0.84), and highest quartile (HR, 0.65; 95% CI: 0.43, 0.99) of daily LPA were associated with lower risks of HF. Higher LPA in the morning, midday, and evening was associated with a higher risk of HF, whereas higher LPA in the night showed an adverse impact on HF risk. These findings were not observed for CHD, stroke, and AF. **Conclusions:** Our findings highlighted that maintaining sufficient duration of LPA might be beneficial for HF as a compensatory PA strategy, but it is also essential to regulate the proportion and timing of LPA.

## Introduction

Light-intensity physical activity (LPA) is characterized by a range of 1.5 to 3 metabolic equivalents of task (METs), representing an energy expenditure of less than threefold the resting metabolic rate [1]. It encompasses activities such as leisurely walking and essential activities of daily living, alongside other forms of physical movement that scarcely impact an individual’s cardiac or respiratory rhythm [1]. LPA constitutes a substantial portion of daily activities [2]. While rapid advancements in industry and technology within contemporary society have systematically removed physical exertion from nearly all aspects of everyday existence, LPA remains the most prevalent component of human movement [2]. Moreover, the available time for moderate- to vigorous-intensity physical activity (MVPA) was substantially reduced due to the demanding pace of work [2,3]. Accumulating evidence has demonstrated that LPA is inversely associated with a reduced risk of various chronic conditions [4–6]. These findings suggest that LPA may serve as a more sustainable and accessible strategy for maintaining cardiovascular health, particularly for individuals who find it challenging to adhere to high-intensity exercise regimens [7]. Therefore, elucidating the effects of LPA on cardiovascular disease (CVD) risk is crucial.

Multiple studies have indicated a protective relationship between physical activity (PA) and CVD, with a particular emphasis on MVPA [8–10]. Large-scale meta-analyses have demonstrated that meeting MVPA recommendations significantly reduces the incidence of coronary heart disease (CHD), stroke, and heart failure (HF) [11]. However, a growing body of evidence suggests that the health benefits of PA may extend into the lower end of the intensity spectrum, which has been traditionally overlooked [11,12]. Often, studies exploring the link between PA and CVD rely on self-reported questionnaires, which can be prone to recall bias and may not accurately capture LPA due to overreporting of more intense activities [13,14]. To address these limitations, using device-based measurements, such as wrist-worn accelerometers, provides a more objective method for assessing PA [13,14]. Evidence from the Objective Physical Activity and Cardiovascular Health (OPACH) in older women study found that higher levels of LPA, measured by accelerometers and adjusted for MVPA, demonstrated protective effects against HF [15]. Furthermore, an analysis of this cohort showed that each additional 1 h/d of engaging in accelerometer-derived LPA was associated with a 14% lower risks of CHD and 8% lower risk of and CVD [15,16]. Similar protective associations have been observed in other cohorts, where LPA was linked to improved cardiometabolic profiles, including reduced systemic inflammation, lower blood pressure, and enhanced insulin sensitivity, all of which are critical pathways in preventing CVD progression [17,18]. These findings suggest that LPA might be a promising way to prevent CVD [15,16]. Despite these promising findings, the current literature remains fragmented. First, most existing studies focus on total LPA volume, leaving the proportion of LPA relative to total daily movement largely unexplored. A high proportion of LPA might paradoxically reflect a physical vulnerability phenotype characterized by the inability to perform MVPA, necessitating a more comprehensive evaluation of the effect of LPA [4]. Second, while the physiological effects of PA are known to interact with the body’s internal clock, evidence regarding the diurnal timing of LPA for CVD prevention is scarce [19,20]. Current guidelines provide little guidance on when during the day LPA is most beneficial, or whether nighttime LPA might conversely indicate disrupted sleep–wake cycles associated with higher risk [21–23]. Moreover, there is a need for large-scale, diverse population studies to validate whether the benefits observed in specific groups can be generalized across broader age ranges and clinical subgroups.

LPA was recommended by the World Health Organization (WHO) to maintain cardiovascular fitness and to compensate for the effect of inadequate MVPA (at least 150 to 300 min/week or equivalents) for inactive populations [1,22,23]. However, more detailed illustrations on the duration, proportion, and timing of LPA were still needed.

Considering LPA constitutes a significant portion of total daily PA, the objective of this study was to assess the associations between accelerometer-derived LPA and the risk of CVD and its subtypes using data from the UK Biobank cohort study. We further explored the relationships between LPA’s duration, proportion, time windows, and CVD risk.

## Methods

### Study design and participants

Data for this study were obtained from the UK Biobank, which is one of the largest open cohorts worldwide. It consisted of half a million participants aged 37 to 73 years who were recruited from 22 assessment centers across England, Wales, and Scotland [24]. Details of this cohort were described elsewhere [24]. Briefly, baseline characteristics and a series of physical examinations were collected and conducted at assessment centers from 2006 to 2010. Participants provided their consent to be followed up for health outcomes. A subgroup of participants with valid email addresses was randomly invited to undergo PA monitoring using a wrist-worn accelerometer for 7 consecutive days from May 2013 to December 2015. The collection of PA data was successfully completed with a total 106,053 participants [25]. The UK Biobank received ethics approval from the North West Multicenter Research Ethics Committee (reference number 11/NW/03820).

In this study, we excluded participants without readable accelerometry data (*n* = 2,393), with less than 3 days of wear, missing data for each 1 h of the 24-h cycle, poor calibration, implausible average acceleration (>100 mg), or without a full week of acceleration data (*n* = 8,178), prevalent CVD before accelerometry test (*n* = 6,311), or with missing values in covariates (*n* = 5,513). A total of 83,658 participants were included in the main analysis, and 89,171 participants were included in the sensitivity analysis with missing imputation methods used for missing values in covariates (Fig. S1).

### Exposure assessment

The data collection utilized the Axivity AX3, a triaxial wrist-mounted accelerometer that serves as the commercial variant of the Open Movement AX3 open-source sensor (https://github.com/digitalinteraction/openmovement). This device was configured to capture triaxial acceleration data over 7 days at 100 Hz with a dynamic range of ±8 g [25,26]. Data processing was conducted using the GGIR open-source package (version 2.4-0, R Foundation), which is the standard validated pipeline for UK Biobank accelerometry [27]. The raw triaxial acceleration data were converted into the Euclidean Norm Minus One (ENMO) metric, which extracts the movement-related acceleration by subtracting local gravity from the vector magnitude, with negative values truncated to zero [25,28]. Accelerometer-measured PA intensity was presented in milligravity units (mg) (1 mg = 0.00981 m/s^2^). The ENMO values were then averaged over 5-s epochs to characterize activity intensity [25,26].

PA intensities were classified based on established ENMO thresholds consistent with recent UK Biobank analyses [29]. LPA was defined as an average acceleration between 30.0 and 100.6 mg. MVPA was defined as acceleration > 100.6 mg (representing activities of ≥ 3 METs). These thresholds were derived from validation studies calibrated against indirect calorimetry [9,30,31]. Activity below 30.0 mg was categorized as either sedentary behavior or sleep, depending on the sustained posture and time of day [27]. The durations of LPA and MVPA per day in this study were derived from the average proportion of time spent doing MVPA or LPA per day [27]. The amount of each category of activity (MET-minutes/week) was calculated by multiplying the energy expended for a specific category of activity (MET: 3.3 for LPA, 4.0 for MPA, and 8.0 for VPA) and the daily duration [9,30,31]. The total amount of all PA was calculated as the sum of the amount of each category of activity (MVPA and LPA). The proportion of LPA of the total PA was calculated by dividing the amount of LPA by the amount of total PA.

The average duration of LPA in each time window across the 24-h cycle was determined by the average proportion of time spent doing LPA per hour across all days [27]. The time windows of LPA were identified using hierarchical clustering analysis on the Pearson correlation coefficients of the average proportions of time spent doing LPA across the 24-h cycle [32]. Then, the duration of LPA in each time window was calculated.

### Outcome assessment

Health outcomes were obtained through the linkage to hospital admission records and death registers. Dates and causes of hospital admissions were identified via records linkage to Health Episode Statistics (England and Wales), the Scottish Morbidity Records (Scotland), and the death registry. Incident CVDs were identified using the International Classification of Diseases 10th edition (ICD-10) codes, with CHD being identified by ICD-10 codes I20 to I25, stroke by ICD-10 codes I60 to I66, atrial fibrillation (AF) by ICD-10 code I48, and HF by ICD-10 codes I42 and I50 [33]. CVD was defined as having any of these diseases.

### Covariates assessment

The regression models accounted for 4 categories of potential confounders. Sociodemographic variables comprised age at the accelerometry test, sex, ethnicity (white or others), education attainment (university degree or other qualifications), and the Townsend deprivation index (TDI). Regarding lifestyle factors, we adjusted for smoking status (current, previous, or never), alcohol drinking frequency (daily or almost daily, 3 or 4 times a week, once or twice a week, 1 to 3 times a month, special occasions only, or never), diet score (from 0 to 5), body mass index (BMI), best hand grip strength, sleep score (low [0 to 1], medium [2–3], and high [4–5]), and quartiles of accelerometer-derived daily MVPA duration. Regarding health history, the hypertension history and self-reported health status (excellent, good, fair, or poor) were adjusted. Furthermore, the season of the accelerometry test was adjusted. The diet quality score was adopted from previously validated methodologies by estimating adherence to the main items of the Mediterranean diet, including higher intakes of vegetables, fruit, and fish and lower intakes of unprocessed red meat and processed meat [34] Additionally, a 5-item self-report scale was utilized to determine sleep quality. Optimal sleep was characterized by an absence of snoring, never or rarely insomnia, infrequent daytime sleepiness, sufficient sleep duration, and ease of waking in the morning [35,36]. For each diet-related or sleep-related item, the participant received a score of 1 if he or she was classified as good quality or 0 if otherwise. The diet or sleep quality scores ranged from 0 to 5, with higher scores indicating higher diet or sleep qualities. The best hand grip strength was defined as the highest of the right- and left-hand values. Hypertension history was determined by self-reported history, health records, and the use of antihypertensive medication.

### Statistical analysis

Descriptive characteristics of all participants according to quartiles of daily LPA duration were presented as means with standard deviation (SD) or medians with interquartile range (IQR) for continuous variables and frequencies with percentages for categorical variables.

Cox proportional hazard regressions were used to estimate the hazard ratios (HRs) and 95% confidence intervals (CIs) of quartiles of LPA level for CVD and its subtypes adjusted for the aforementioned covariates. We tested 3 models: (a) Model 1 was adjusted for age, sex, and ethnicities; (b) Model 2 was adjusted for Model 1 plus educational level, TDI, smoking status, alcohol drinking frequency, BMI, best hand grip strength, diet score, sleep score, self-reported health status, history of hypertension, and season of accelerometry test; and (c) Model 3 was adjusted for Model 2 plus MVPA level. The follow-up duration started at the date of the accelerometry test and ended with the first date of the outcome of interest, the date of death, or the end of follow-up (2022 Sep 31), whichever came first. Proportional hazard assumptions were tested through a visual examination of the Schoenfeld residuals. To evaluate the potential nonlinear associations between daily LPA duration, proportion of LPA, and LPA duration across different time windows with incident CVD, we employed generalized additive models (GAMs) with thin plate regression splines as the smoothing basis. The optimal degree of smoothness for each spline term was determined by minimizing the restricted maximum likelihood criterion. To prevent overfitting, we monitored the effective degrees of freedom for each smooth term, ensuring that the complexity of the curves was statistically supported by the number of incident events. Furthermore, to test the structural stability of the identified nonlinear patterns, we conducted a sensitivity analysis using restricted cubic splines (RCS) with 4 knots.

To assess the robustness of the associations and identify potential high-risk populations, we conducted stratified analyses by key baseline characteristics, including age groups (<60 and ≥60 years), sex (males and females), levels of measured MVPA (high and low PA level defined using the PA guideline of WHO [150 min/week] as the cutoff point), BMI (<25 or ≥25 kg/m^2^), and prevalent hypertension status at baseline. Furthermore, to explore whether the protective effect of LPA is modified by genetic susceptibility, we performed a stratified analysis based on the polygenic risk score (PRS) for CVD. The statistical significance of interactions between LPA metrics and stratification variables was evaluated by including an interaction term in the Cox proportional hazards models. The genetic susceptibility to CVD was quantified using the standardized PRS obtained from the UK Biobank (Field 26223). This PRS was derived using a validated genome-wide approach that integrates millions of risk-associated genetic variants specifically for circulatory system diseases [37]. For the stratified analysis, we standardized the raw PRS and further categorized participants into low and high genetic risk groups based on the median value of the PRS distribution within our study population.

To estimate the theoretical effect of reallocating time from sedentary behavior, sleep, or MVPA to LPA while keeping the total 24-h time constant, we employed isotemporal substitution models. In these models, a specific activity component was omitted from the model that included all other activity types and the total 24-h time. The regression coefficients for the remaining activity types represent the estimated effect of substituting a fixed duration (10, 20, or 30 min/d) of the omitted activity with an equivalent amount of LPA. HRs and 95% CIs were calculated for each reallocation increment to assess the potential clinical impact of time-use displacement on incident CVD risk.

In sensitivity analysis, we reran the analyses among participants with missing values in covariates imputed using multiple imputations with 5 replications based on a chained equation method. Additionally, 2-year landmark analyses were adopted by excluding participants who developed events of interest within 2 years after the accelerometer assessment to avoid reverse causal relationships.

Statistical analyses were conducted using R, version 4.2.0 (the R Foundation for Statistical Computing). All tests were 2-sided with a significance level of 0.05.

## Results

### Study participants and population characteristics

A total of 83,658 participants were included in the main analysis. The analysis sample comprised 35,050 (41.9%) men, with a mean age of 62.02 (±7.83) years at the accelerometry test. The characteristics of participants categorized by quartiles of LPA level were presented in Table 1. Among all, participants in the lowest quartile of LPA exhibited a higher likelihood of being male, white ethnicity, higher educated, less deprived, current smokers, with higher BMI, higher best hand grip strength, lower self-reported heath status, lower diet scores, lower sleep scores, and higher probabilities of hypertension.

**Table 1.** Characteristics of study participants by quartiles of LPA level. The cutoff points of quartiles of LPA were 3.94, 4.94, and 6.10 h/d.

Characteristics	Overall (*n* = 83,658)	Quartiles of LPA level			
		Q1 (*n* = 20,901)	Q2 (*n* = 20,945)	Q3 (*n* = 21,178)	Q4 (*n* = 20,634)
Age at accelerometry test, years, mean (SD)	62.02 (7.83)	61.82 (8.02)	62.16 (7.80)	62.38 (7.72)	61.73 (7.76)
Men, *n* (%)	35,050 (41.9)	12,623 (60.4)	9,379 (44.8)	7,339 (34.7)	5,709 (27.7)
White, *n* (%)	81,223 (97.1)	20,352 (97.4)	20,419 (97.5)	20,550 (97.0)	19,902 (96.5)
University, *n* (%)	37,123 (44.4)	10,320 (49.4)	9,744 (46.5)	9,100 (43.0)	7,959 (38.6)
TDI, median (IQR)	−2.47 [−3.83, −0.24]	−2.33 [−3.75, 0.18]	−2.49 [−3.85, −0.30]	−2.57 [−3.88, −0.43]	−2.50 [−3.85, −0.38]
Smoking status, *n* (%)					
Never	48,600 (58.1)	12,028 (57.5)	12,038 (57.5)	12,263 (57.9)	12,271 (59.5)
Previous	29,405 (35.1)	7,189 (34.4)	7,504 (35.8)	7,614 (36.0)	7,098 (34.4)
Current	5,653 (6.8)	1,684 (8.1)	1,403 (6.7)	1,301 (6.1)	1,265 (6.1)
Alcohol intake frequency, *n* (%)					
Daily or almost daily	19,145 (22.9)	4,875 (23.3)	4,953 (23.6)	4,778 (22.6)	4,539 (22.0)
Three or four times a week	21,952 (26.2)	5,511 (26.4)	5,582 (26.7)	5,638 (26.6)	5,221 (25.3)
Once or twice a week	21,115 (25.2)	5,221 (25.0)	5,317 (25.4)	5,457 (25.8)	5,120 (24.8)
One to three times a month	9,175 (11.0)	2,284 (10.9)	2,278 (10.9)	2,269 (10.7)	2,344 (11.4)
Special occasions only	7,735 (9.2)	1,881 (9.0)	1,794 (8.6)	1,924 (9.1)	2,136 (10.4)
Never	4,536 (5.4)	1,129 (5.4)	1,021 (4.9)	1,112 (5.3)	1,274 (6.2)
BMI, kg/m^2^, mean (SD)	26.58 (4.48)	27.65 (4.90)	26.76 (4.39)	26.25 (4.25)	25.67 (4.11)
Best hand grip strength, kg, mean (SD)	33.04 (10.99)	36.07 (11.29)	33.57 (11.01)	31.85 (10.66)	30.67 (10.19)
Self-reported health status, *n* (%)					
Excellent	19,127 (22.9)	4,408 (21.1)	4,902 (23.4)	5,035 (23.8)	4,782 (23.2)
Good	50,837 (60.8)	12,092 (57.9)	12,809 (61.2)	13,051 (61.6)	12,885 (62.4)
Fair	11,987 (14.3)	3,703 (17.7)	2,836 (13.5)	2,775 (13.1)	2,673 (13.0)
Poor	1,707 (2.0)	698 (3.3)	398 (1.9)	317 (1.5)	294 (1.4)
Diet score, *n* (%)					
0	2,058 (2.5)	710 (3.4)	525 (2.5)	429 (2.0)	394 (1.9)
1	9,847 (11.8)	3,161 (15.1)	2,473 (11.8)	2,183 (10.3)	2,030 (9.8)
2	19,885 (23.8)	5,648 (27.0)	5,039 (24.1)	4,796 (22.6)	4,402 (21.3)
3	24,761 (29.6)	5,982 (28.6)	6,290 (30.0)	6,318 (29.8)	6,171 (29.9)
4	18,624 (22.3)	3,806 (18.2)	4,641 (22.2)	5,039 (23.8)	5,138 (24.9)
5	8,483 (10.1)	1,594 (7.6)	1,977 (9.4)	2,413 (11.4)	2,499 (12.1)
Sleep score, *n* (%)					
Low (0–1)	8,711 (10.4)	2,599 (12.4)	2,133 (10.2)	2,046 (9.7)	1,933 (9.4)
Moderate (2–3)	47,174 (56.4)	12,057 (57.7)	11,882 (56.7)	11,880 (56.1)	11,355 (55.0)
High (4–5)	27,773 (33.2)	6,245 (29.9)	6,930 (33.1)	7,252 (34.2)	7,346 (35.6)
Hypertension, *n* (%)	18,940 (22.6)	5,693 (27.2)	4,862 (23.2)	4,452 (21.0)	3,933 (19.1)
Season of accelerometry test, *n* (%)					
Spring	19,118 (22.9)	4,658 (22.3)	4,858 (23.2)	4,912 (23.2)	4,690 (22.7)
Summer	21,828 (26.1)	5,217 (25.0)	5,301 (25.3)	5,520 (26.1)	5,790 (28.1)
Autumn	24,914 (29.8)	6,304 (30.2)	6,231 (29.7)	6,332 (29.9)	6,047 (29.3)
Winter	17,798 (21.3)	4,722 (22.6)	4,555 (21.7)	4,414 (20.8)	4,107 (19.9)
LPA, h/d, median (IQR)	4.94 [3.94, 6.10]	3.26 [2.78, 3.63]	4.46 [4.22, 4.70]	5.49 [5.21, 5.79]	6.99 [6.51, 7.75]
MVPA, h/d, median (IQR)	0.55 [0.27, 0.96]	0.51 [0.24, 0.96]	0.58 [0.27, 1.03]	0.58 [0.31, 0.96]	0.55 [0.27, 0.93]

SD, standard deviation; IQR, interquartile range; TDI, Townsend deprivation index; BMI, body mass index; LPA, light physical activity; MVPA, moderate-to-vigorous physical activity

### Associations between LPA and risk of CVDs

Over a median follow-up duration of 7.98 (IQR: 7.43 to 8.47) years, a total of 2,239 CVDs, 1,014 CHDs, 352 strokes, 796 AFs, and 194 HFs were recorded. In the multivariable-adjusted model, we observed a significant inverse association between LPA and the risk of HF across all quartiles of LPA. Compared to participants with the lowest level of LPA (≤3.94 h/d), participants in the second (3.94 to ≤4.94 h/d), third (4.94 to ≤6.10 h/d), and fourth (>6.10 h/d) quartiles of LPA levels had a significantly lower risk of HF by 36% (95% CI: 0.44 to 0.93), 44%, (95% CI: 0.37 to 0.84), and 35% (95% CI: 0.43 to 0.99) respectively, as shown in Table 2. Furthermore, we observed an effect modification by age on the association between LPA and HF risk. The beneficial inverse associations of LPA with HF appeared stronger in participants aged above 60 years, indicating that older individuals may derive greater benefits from equivalent levels of LPA (*P* for multiplicative interaction = 0.013, Fig. S2). Meanwhile, participants who engaged in less than 150 min/week of MVPA exhibited a larger risk reduction in HF relative to their counterparts (*P* for multiplicative interaction = 0.010), thereby suggesting potential benefits for participants who were unable to meet the WHO’s guideline. Similar patterns of interaction were observed for BMI level and hypertension status. The protective effect of LPA was significantly stronger among participants with overweight or obesity (*P* for multiplicative interaction = 0.018) and those with preexisting hypertension (*P* for multiplicative interaction = 0.024). Although no significant effect modification was found for sex or PRS categories, the absolute risk reduction was more pronounced in individuals with high genetic risk. No statistically significant associations were found between LPA and CVD, CHD, stroke, and AF. The main results were robust in 2 sensitivity analyses.

**Table 2.** Association of LPA with the risk of cardiovascular disease. The cutoff points of quartiles of LPA were 3.94, 4.94, and 6.10 h/d.

Outcomes	Quartiles of LPA	Events	Crude incidence rate	Hazard ratio (95% confidence interval)		
			(per 10,000 person-years)	Model 1^a^	Model 2^b^	Model 3^c^
CVD	Q1	646	39.92	Reference	Reference	Reference
	Q2	552	33.80	0.93 (0.83, 1.04)	0.93 (0.83, 1.04)	0.93 (0.83, 1.04)
	Q3	541	32.69	0.96 (0.86, 1.08)	0.96 (0.85, 1.08)	0.96 (0.85, 1.08)
	Q4	500	30.95	1.01 (0.90, 1.14)	1.01 (0.89, 1.14)	1.01 (0.89, 1.14)
CHD	Q1	311	19.12	Reference	Reference	Reference
	Q2	249	15.16	0.90 (0.76, 1.07)	0.91 (0.77, 1.08)	0.91 (0.77, 1.08)
	Q3	249	14.95	0.98 (0.82, 1.16)	0.98 (0.83, 1.17)	0.99 (0.83, 1.18)
	Q4	205	12.61	0.93 (0.77, 1.11)	0.93 (0.77, 1.12)	0.94 (0.78, 1.13)
Stroke	Q1	93	5.69	Reference	Reference	Reference
	Q2	82	4.97	0.93 (0.69, 1.26)	0.95 (0.70, 1.28)	0.94 (0.70, 1.27)
	Q3	91	5.45	1.08 (0.80, 1.44)	1.11 (0.82, 1.49)	1.10 (0.81, 1.48)
	Q4	86	5.27	1.14 (0.84, 1.55)	1.18 (0.87, 1.61)	1.18 (0.86, 1.60)
AF	Q1	212	13.02	Reference	Reference	Reference
	Q2	208	12.66	1.04 (0.86, 1.26)	1.00 (0.83, 1.22)	1.00 (0.82, 1.21)
	Q3	189	11.35	0.98 (0.80, 1.20)	0.93 (0.76, 1.14)	0.93 (0.76, 1.14)
	Q4	187	11.51	1.09 (0.89, 1.34)	1.04 (0.85, 1.28)	1.04 (0.85, 1.28)
HF	Q1	83	5.08	Reference	Reference	Reference
	Q2	43	2.61	0.55 (0.38, 0.80)	0.63 (0.43, 0.92)	0.64 (0.44, 0.93)
	Q3	34	2.03	0.46 (0.30, 0.68)	0.55 (0.36, 0.83)	0.56 (0.37, 0.84)
	Q4	34	2.08	0.52 (0.34, 0.78)	0.64 (0.42, 0.98)	0.65 (0.43, 0.99)

LPA, light physical activity; CVD, cardiovascular disease; CHD, coronary heart disease; AF, atrial fibrillation; HF, heart failure

^a^ Model 1 adjusted for age, sex, and ethnicities.

^b^ Model 2 additionally adjusted for educational level, Townsend deprivation index, smoking status, alcohol drinking frequency, body mass index, best hand grip strength, self-reported health status, diet quality, sleep quality, hypertension, and season of accelerometry test, besides covariates in model 1.

^c^ Model 2 additionally adjusted for moderate-to-vigorous physical activity level, besides covariates in model 2.

Isotemporal substitution analysis revealed that replacing sedentary behavior with LPA was associated with a significant reduction in the risk of incident HF (Table S1). Reallocating 10, 20, or 30 min/d of sedentary time to LPA was associated with a 3% (HR, 0.97; 95% CI: 0.96 to 0.99), 5% (HR, 0.95; 95% CI: 0.91 to 0.98), and 8% (HR, 0.92; 95% CI: 0.87 to 0.97) lower risk of HF, respectively. In contrast, substituting sleep with LPA did not yield statistically significant changes in HF risk.

**Fig. 1.**
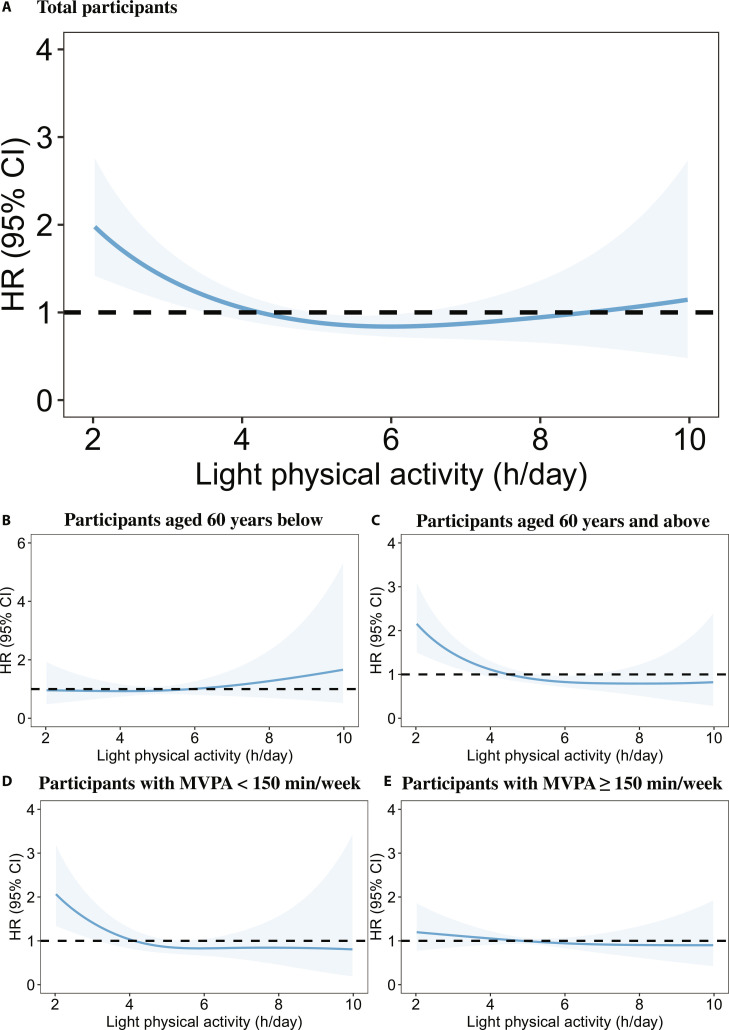
Nonlinear association of LPA on incident heart failure. Abbreviations: HR, hazard ratio; CI, confidence interval; MVPA, moderate-to-vigorous physical activity. Adjusted for age, sex, ethnicities, educational level, Townsend deprivation index, smoking status, alcohol drinking frequency, body mass index, best hand grip strength, self-reported health status, diet quality, sleep quality, hypertension, and season of accelerometry test. The effective degrees of freedom were 2.706, 1.361, 2.458, 2.364, and 1.241 for splines in (A) to (E), respectively.

**Fig. 2.**
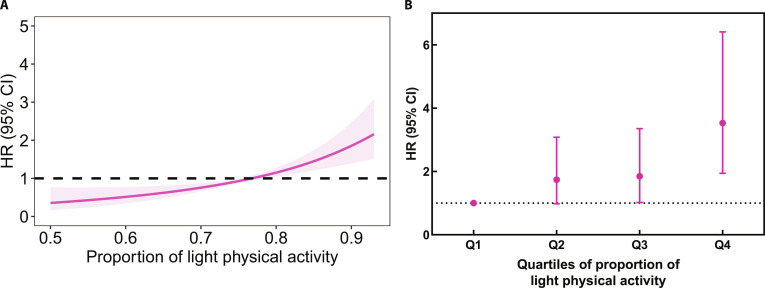
Nonlinear association of the proportion of LPA on incident heart failure. Abbreviations: HR, hazard ratio; CI, confidence interval. (A) The nonlinear association of proportion of LPA with incident HF. (B) The association of quartiles of proportion of LPA with incident HF. Adjusted for age, sex, ethnicities, educational level, Townsend deprivation index, smoking status, alcohol drinking frequency, body mass index, best hand grip strength, self-reported health status, diet quality, sleep quality, hypertension, and season of accelerometry test. The effective degrees of freedom of splines for proportion of LPA were 1.294.

An L-shaped relationship was found between the duration of LPA and the risk of HF (Fig. 1). These nonlinear associations were more profound among participants aged ≥60 years and those with MVPA < 150 min/week. An increased risk of HF was observed when LPA accounts for higher proportion of the total PA (reversed L-shaped) (Fig. 2). Compared with participants in the lowest quartile of proportion of LPA, those in the third (HR, 1.85; 95% CI: 1.02 to 3.35) and highest (HR, 3.53; 95% CI: 1.94 to 6.41) quartile had higher risks of HF.

### Timing of LPA and CVDs

**Fig. 3.**
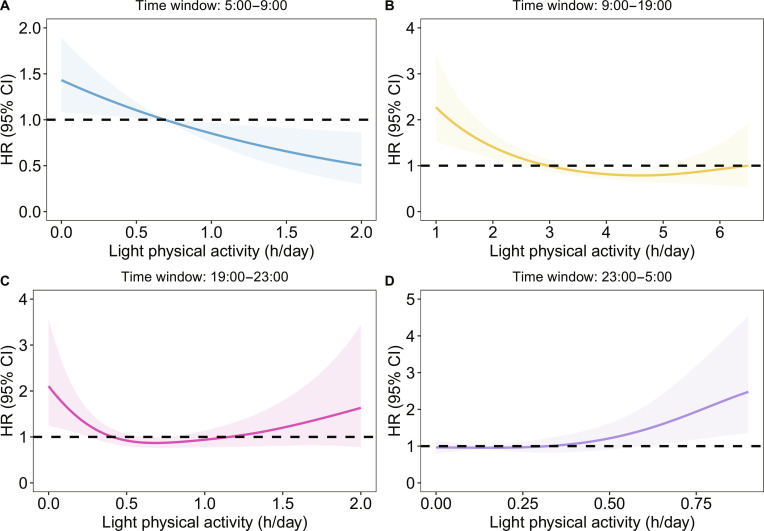
Nonlinear association of LPA within 4 time windows with incident heart failure. Abbreviations: HR, hazard ratio; CI, confidence interval. Panels (A), (B), (C), and (D) were the nonlinear associations of LPA with the risk of heart failure in the time window of morning (5:00 to 9:00), daytime (9:00 to 19:00), evening (19:00 to 23:00), and night (23:00 to 5:00), respectively. Adjusted for age, sex, ethnicities, educational level, Townsend deprivation index, smoking status, alcohol drinking frequency, body mass index, best hand grip strength, self-reported health status, diet quality, sleep quality, hypertension, and season of accelerometry test. The effective degrees of freedom were 1.001, 2.522, 3.177, and 2.948 for splines in (A) to (D), respectively.

The hierarchical clustering analysis identified 4 distinct time windows (Fig. S3) for further analyses. Figure S4 presents the distributions of LPA within these identified time windows. As depicted in Fig. 3**,** maintaining an LPA duration of ≥0.53 h in the morning (5:00 to 9:00), ≥3 h during midday (9:00 to 19:00), ≥0.48 h in the evening (19:00 to 23:00), and <0.5 h at night (23:00 to 5:00) is beneficial for reducing the risk of HF. The results were similar among participants aged <60 years and ≥60 years, while the associations of LPA with HF risk in 4 time windows were more statistically significant among those aged ≥60 years (Figs. S5 and S6).

## Discussion

Using data from a large cohort study, this study provides valuable evidence on LPA for CVD protection. According to our study, higher daily LPA duration was associated with a lower risk of HF while higher proportion of LPA out of the total PA was positively related to the HF risk. L-shaped and reversed L-shaped nonlinear association were observed for the relationships of LPA duration and LPA proportion with the risk of HF. As for the time windows, higher LPA duration in the morning (5:00 to 9:00), higher LPA duration in midday (9:00 to 19:00), higher LPA duration evening (19:00 to 23:00), and lower LPA duration in the night (23:00 to 5:00) were associated with a lower risk of HF.

### Comparison with other studies

Consistent with our results, evidence from the OPACH in Older Women study showed that per SD higher accelerometer-measured LPA was associated with 12% and 20% lower risks of overall HF and HF with preserved ejection fraction, respectively [15]. Although this study provides valuable evidence that even LPA conveys improved cardiovascular health, the constraints of the study population (older women) might compromise the generalization of the results [12]. However, more studies on PA with HF mainly focused on the effects of MVPA, and the recommendations of the duration, proportion, and especially timing of LPA for HF prevention were not specified [29,38,39]. Our study extended current studies and provided valuable evidence on the effects of LPA with HF. LPA constitutes the majority of the time of total daily living PA among adults [40]. Moreover, the increase of LPA is more achievable than MVPA [41]. It is essential to understand the consequences of LPA [16]. Our study specified the amount of LPA for HF prevention, which is that more than 4.2 h of LPA per day and less than 78% proportion of LPA are beneficial for HF. However, the observed increase in HF risk associated with an extremely high proportion of LPA should be interpreted with caution. As noted by our analysis, a high relative proportion of LPA may inherently reflect a phenotype of limited MVPA or reduced physical reserve rather than a direct harmful effect of LPA itself. Our results confirmed that LPA’s protective role is most prominent in those not meeting MVPA guidelines, suggesting that for individuals with low exercise capacity, LPA serves as a critical compensatory strategy. The effect of LPA with HF was modified by age group, MVPA level, BMI level, and hypertension status according to our results. The consequence of LPA on HF was more profound among participants aged 60 years and above, those below the recommended MVPA level, overweight/obese individuals, and those with hypertension. Furthermore, our study found that the inverse association between LPA and HF remained consistent across different genetic risk levels for CVD. LPA is a more feasible alternative for increasing PA for older adults and those who could not perform MVPA due to certain conditions [42]. Our isotemporal substitution analysis suggested that reallocating as little as 10 to 30 min/d from sedentary behavior to LPA was associated with a significant reduction in HF risk, reinforcing the potential public health impact of lifestyle displacements.

Moreover, recent studies have found that exercise timing is another important influence on health outcomes due to the circadian rhythm of humans [19,20,42]. Albalak et al. [20] found that morning PA was associated with lower risks of CVD, but this study did not consider the timing of PA across different intensities. Our study contributes to determining the proper LPA duration across different time windows. The association of LPA duration and HF risk differed by time windows. In the midday (9:00 to 19:00) and evening (19:00 to 23:00) periods, L-shaped association of LPA with HF was observed with LPA duration > 3 h (>30% of the total time span) and >0.48 h (12% of the total time span) showed a potential association with a lower risk of HF. In the morning period (5:00 to 9:00), higher LPA was inversely associated with the risk of HF, indicating that more LPA duration might be considered for HF prevention. The specified LPA duration in different time windows should be viewed as illustrative of a high-activity phenotype rather than prescriptive clinical targets. Given the observational nature of the UK Biobank data, these thresholds require validation in independent, intervention-based studies. The observed association between higher nighttime LPA (23:00 to 5:00) and increased HF risk should be interpreted as a potential clinical marker rather than a modifiable behavioral target. Increased nocturnal activity may reflect sleep-disruptive conditions, such as nocturia or paroxysmal nocturnal dyspnea, which are known prodromal symptoms of HF. Therefore, rather than suggesting a reduction in night activity, our findings highlight the importance of monitoring nocturnal restlessness as a proxy for underlying cardiovascular vulnerability.

Our study showed that there was no significant association with total CVD (CHD, stroke, and AF). An analysis of the OPACH study has suggested that daily life movement (defined as standing and moving in a confined space such as performing housework or gardening) and LPA were inversely associated with CVD risk [16,40]. The inconsistencies among results may be due to differences in the characteristics of participants, exposure definition, covariate adjustment, sample size, and others. Further studies on these associations are needed.

### Underlying mechanisms

Evidence from mechanistic experiments indicates that brief but recurrent intervals of LPA during the day can dampen postprandial glycemic and insulinemic responses. Furthermore, compared to prolonged sedentary behavior, LPA has been shown to lower pro-inflammatory lipid profiles while elevating lipids linked to antioxidant potential [18,43]. Targeted LPA interventions may also mitigate adiposity and optimize blood pressure levels [2,18,44]. From a physiological perspective, arterial stiffness—a hallmark of vascular senescence—is considered a pivotal intermediate and a known contributor to HF. Breaking up sitting time with LPA might prevent the progression of arterial stiffening, a condition often exacerbated by adverse hemodynamic shifts resulting from venous pooling [45]. One possible reason for the favorable timing of LPA in the morning, daytime, and evening could be related to the circadian modulation of cardiometabolic reactivity [46]. The unfavorable timing of LPA in the night might be due to the effects of the disruption of circadian rhythms on reperfusion injury [46]. More specific underlying mechanisms remain to be elucidated.

### Implications for public health

LPA increasing and replacing sedentary behavior with LPA is more achievable than MVPA in PA intervention programs [41]. Among older adults and people incapable of MVPA, LPA is an important alternative for PA [12]. Thus, public health suggestions on LPA are essential. Moreover, the favorable timings of LPA are morning (5:00 to 9:00), midday (9:00 to 19:00), and evening (19:00 to 23:00). LPA at night (23:00 to 5:00) was linked to higher risk of HF. Notably, the interpretation of nighttime LPA requires caution. Unlike daytime activity, which is typically a modifiable behavioral exposure, nighttime LPA may serve as a clinical marker of sleep disturbances or prodromal symptoms of HF, such as paroxysmal nocturnal dyspnea or orthopnea. Increased movement during rest hours often reflects poor sleep quality or nocturnal discomfort, both of which are independent predictors of adverse cardiovascular outcomes. Therefore, the observed associations for nighttime LPA might partly represent reverse causation, where preexisting subclinical HF manifestations manifest as increased physical restlessness during the night. Although we employed a 2-year landmark analysis to mitigate this bias, the possibility that nocturnal activity reflects underlying health status rather than a lifestyle choice cannot be entirely ruled out.

### Strength and limitations

To our knowledge, this is one of the few studies to comprehensively assess the effect of LPA’s duration, proportion, and timing for CVD prevention using a large-scale prospective design. Using data from the accelerometry tests, LPA in this study was more accurately measured than self-report due to recall bias. With the large sample size of the UK Biobank, this study offered strong suggestions on the association of LPA with CVD. Moreover, 2 sensitivity analyses and the inclusion of multiple covariates strengthened the robustness of the results. However, there were some limitations in this study. First, many covariates in this study were collected at the baseline visit, which, in some cases, was several years before accelerometry tests. However, studies on the association of PA with health outcomes using the UK Biobank revealed that covariates mentioned above had steady influence [9,19,27,38,39,42,47] Second, the cohort within the UK Biobank exhibits a more favorable health profile compared to the broader public, which might cause healthy volunteer bias. However, studies showed that this lack of representativeness does not impact the valid and generalizable assessments of associations [48]. Thirdly, only a fifth of the participants in the UK Biobank underwent accelerometry tests, potentially introducing selection bias. Notwithstanding, this study still provided crucial evidence on the association of LPA with CVD, leveraging a large sample size from the UK Biobank with accelerometry data. Fourth, the representativeness of a 7-day accelerometer measurement for longer-term behaviors remains uncertain. Furthermore, the time windows in our study were derived empirically through clustering analysis. While this approach captures the data-driven patterns of this specific cohort, it may limit the direct generalizability of the exact hourly boundaries to other populations with different socio-cultural or occupational routines. It is important to note that the specific thresholds identified in our study (4.2 h/d of LPA or a 78% LPA proportion) were derived empirically from spline models within the UK Biobank cohort. While these values provide a quantitative framework to characterize the dose–response relationship between LPA and HF risk in this large-scale population, they should be interpreted as illustrative of a low-risk activity phenotype rather than absolute clinical targets. The generalizability of these precise cut-points to other populations remains to be validated. Future independent studies are needed to determine if these specific thresholds are consistent across diverse global cohorts. Moreover, although traditional risk factors and potential confounders were all adjusted in the regression models, residual confounding of unmeasured factors still could not be fully ruled out. Because of the observational nature of this study, the identified associations between LPA timing and HF risk do not imply direct causality. While we observed specific time-window effects, these patterns may be influenced by unmeasured lifestyle factors or underlying biological rhythms, and our findings should be considered as hypothesis-generating rather than definitive clinical prescriptions. Further studies on these aspects are needed.

## Conclusion

In this large-scale cohort, we identified significant nonlinear associations between accelerometer-measured LPA and the risk of incident HF. Specifically, increased daily LPA duration exhibited an L-shaped relationship with lower HF risk, whereas an excessively high proportion of LPA within total PA was associated with an elevated risk. Higher LPA during the morning, midday, and evening was associated with reduced HF risk, while increased activity during the night may serve as a clinical marker of underlying sleep disturbances or prodromal symptoms. These findings suggest that maintaining sufficient duration of LPA might be beneficial for HF as a compensatory PA strategy, especially for older adults (>60 years), individuals not meeting the WHO-recommended MVPA levels, and those with preexisting hypertension or overweight/obesity. However, given the observational nature of these data, further validation in clinical intervention trials were needed.

## Ethical Approval

UKB received ethical approval from the National Information Governance Board for Health and Social Care and the National Health Service North West Multi-Center Research Ethics Committee. All participants gave informed consent through electronic signature before enrollment in the study. Analyses were conducted under application number 79095. This research conforms to the Declaration of Helsinki.

## Data Availability

The UK Biobank resource is open to all researchers (https://www.ukbiobank.ac.uk). Statistical code is available on request via email from the corresponding authors.
