# Supplementary material for: Association of Accelerometer-Measured Light-Intensity Physical Activity with Cardiovascular Diseases: A Large Cohort Study
Source: Health Data Sci. 2026 Aug 12;6:0470. doi: 10.34133/hds.0470 (PMC13462243; doi:10.34133/hds.0470)
Supplement: Supplementary file 1 [file hds.0470.f1.docx]

**Supplementary material**

UK Biobank participants performed accelerometry tests

N = 106,053

N = 103,660

**Excluded:**

2393 without readable accelerometry data;

N = 95,482

**Excluded:**

11 with poor calibration;

6994 with < 3 days of wear, absent data in each one-hour period of the 24-hour cycle;

15 with implausible average acceleration (> 100 milli-g);

1158 without a full week of acceleration data.

**Excluded** 6311 with prevalent cardiovascular disease.

N = 89,171

included in sensitivity analysis

**Excluded** 5513 participants without missing values in covariates.

N = 83,658 included in

main analysis

**Supplemental figure 1** Participant selection flowchart.

| (a) | **(b)** |
| --- | --- |
|  |  |

**Supplemental figure 2 N**on-linear association of LPA on incident heart failure using restricted cubic spline models.
Abbreviation: HR, hazard ratio. CI, confidence interval. MVPA, moderate-to-vigorous physical activity.

Adjusted for age, sex, ethnicities, educational level, Townsend deprivation index, smoking status, alcohol drinking frequency, body mass index, best hand grip strength, self-reported health status, diet quality, sleep quality, hypertension, and season of accelerometry test.

**Supplemental figure 3** Subgroup and sensitivity analysis of LPA and heart failure.

The cut-off points of quartiles of LPA were 3.94, 4.94, and 6.10 h/day.

Abbreviation: HR, hazard ratio. CI, confidence interval. MVPA, moderate-to-vigorous physical activity.

Adjusted for age, sex, ethnicities, educational level, Townsend deprivation index, smoking status, alcohol drinking frequency, body mass index, best hand grip strength, self-reported health status, diet quality, sleep quality, hypertension, and season of accelerometry test.

**Supplemental table 1 Estimated HRs (95% CI) for incident heart failure associated with the isotemporal substitution of LPA for others.**

| **Activities replaced by LPA** | **Reallocation duration** | **HR (95% CI)** | **P-value** |
| --- | --- | --- | --- |
| **Sedentary Behavior** | 10 minutes/day | 0.97 (0.96, 0.99) | 0.001 |
|  | 20 minutes/day | 0.95 (0.91, 0.98) | 0.001 |
|  | 30 minutes/day | 0.92 (0.87, 0.97) | 0.001 |
| **Sleep** | 10 minutes/day | 0.99 (0.97, 1.01) | 0.476 |
|  | 20 minutes/day | 0.98 (0.94, 1.03) | 0.476 |
|  | 30 minutes/day | 0.98 (0.92, 1.04) | 0.476 |
| **MVPA*** | 10 minutes/day | 1.01 (0.96, 1.06) | 0.734 |
|  | 20 minutes/day | 1.02 (0.92, 1.13) | 0.734 |
|  | 30 minutes/day | 1.03 (0.88, 1.20) | 0.734 |

Abbreviation: HR, hazard ratio. CI, confidence interval. MVPA, moderate-to-vigorous physical activity.

All models were adjusted for total 24-hour time, age, sex, race, education, BMI, smoking status, alcohol consumption, diet score, sleep duration, prevalent chronic conditions, season of wear, self-rated health, and grip strength. *Replacing MVPA with LPA is expected to show an increased risk (HR > 1.00), serving as a biological consistency check.

**Supplemental figure 4** Correlation of 24-hour LPA.

The color of each cube indicates the value of the correlation coefficient between two hours.

“o” indicated that this correlation coefficient was not statistically significant after Bonferroni adjustment (0.05/276).

**Supplemental figure 5** Distributions of LPA in four time-windows.

|  |  |
| --- | --- |
|  |  |

**Supplemental figure 6** Non-linear association of LPA within four time-windows with incident heart failure among participants aged 60 years below.

Abbreviation: HF, heart failure.

Figure (a), (b), (c), and (d) were the non-linear associations of LPA with the risk of heart failure in the time window of morning (5:00~9:00), daytime (9:00~19:00), evening (19:00~23:00) and night (23:00~5:00), respectively.

Adjusted for age, sex, ethnicities, educational level, Townsend deprivation index, smoking status, alcohol drinking frequency, body mass index, best hand grip strength, self-reported health status, diet quality, sleep quality, hypertension, and season of accelerometry test

|  |  |
| --- | --- |
|  |  |

**Supplemental figure 7** Non-linear association of LPA within four time-windows with incident heart failure among participants aged 60 years and above.

Abbreviation: HF, heart failure.

Figure (a), (b), (c), and (d) were the non-linear associations of LPA with the risk of heart failure in the time window of morning (5:00~9:00), daytime (9:00~19:00), evening (19:00~23:00) and night (23:00~5:00), respectively.

Adjusted for age, sex, ethnicities, educational level, Townsend deprivation index, smoking status, alcohol drinking frequency, body mass index, best hand grip strength, self-reported health status, diet quality, sleep quality, hypertension, and season of accelerometry test
